# Experimental study on axial compression for composite hollow column of steel fiber, high-strength lightweight aggregate concrete and angle steel

**DOI:** 10.1038/s41598-022-16581-w

**Published:** 2022-07-20

**Authors:** Zehui Xiang, Dan Qiao, Jiangang Niu, Weiheng Liu

**Affiliations:** 1 School of Civil Engineering, Inner Mongolia University of Science and Technology, Baotou, 014010 Inner Mongolia China; 2 School of Civil Engineering, Beijing University of Technology, Beijing, 100029 China

**Keywords:** Engineering, Civil engineering

## Abstract

In order to study the axial compression performance for composite hollow column of steel fiber, high-strength lightweight aggregate concrete and angle steel, the axial compression tests were carried out on five composite hollow columns of steel fiber, high-strength lightweight aggregate concrete and angle steel with the variation parameters of hollow ratio (0%, 15%, 16%, 32% and 36%) and the form of opening (round hole and square hole). The failure phenomena and failure forms of the specimens were observed, and their stress–strain curves were measured, and the axial bearing capacity formula suitable for composite hollow column of steel fiber, high-strength lightweight aggregate concrete and angle steel was established. The following conclusions may be obtained from the test results: the axial compression performance for the composite hollow columns of angle steel is influenced by the hollow ratio and opening form greatly. The axial compression performance for composite hollow columns of steel fiber, high-strength lightweight aggregate concrete and angle steel is almost close to that of composite solid columns when the hollow ratio is low; The higher the void ratio, the more the cracks at the surface of the concrete, some transverse cracks appear, the peak load decreases by about 5–38%, and the deformation ductility coefficient increases gradually; The deformation ductility coefficient of round-hole hollow column is lower than that of square-hole hollow column. Based on the test, the finite element software ABAQUS is used to simulate the SCAH column. The correctness of the model is verified via the comparison between the numerical simulation results and the test results. At the same time, the stress nephogram of concrete and steel at different stages and the stress nephogram at concrete restraint state are simulated. According to the finite element simulation results, Mander model is used to calculate the axial compression bearing capacity of composite hollow column of angle steel. The high calculation accuracy and suitable for popularization can be obtained.

## Introduction

Along with the development of high-rise and long-span buildings, the structural weight of buildings is becoming larger and larger. The research shows that reducing the own weight of buildings can greatly reduce the impact of strong earthquakes on buildings, the amount of materials and the project cost, so as to obtain sustainable environmental economic benefits and environmental social benefits^1,2^. On the one hand, the high-performance concrete shall be used to minimize the own weight of the building. The structure may be reduced effectively via replacing the ordinary concrete with the high-strength lightweight aggregate concrete at the same strength grade^3–5^. However, the brittleness of concrete increases significantly along with the gradual increase of the strength grade of high-strength lightweight aggregate concrete^6^. It can be found that the crack resistance, ductility energy consumption and the brittleness may be improved effectively by adding the steel fiber to the lightweight aggregate concrete^7^. On the other hand, it is to optimize the structural form and change the beam column section. It is found that the hollow column with holes at the column section can effectively reduce the own weight of the structure^8,9^. The hollow column is widely used in the building structure and pier due to small own weight, huge anti-bending and torsional stiffness.

In recent years, a series of researches on the axial compression performance of hollow column via the changes of hollow ratio and section form have been conducted by the scholars at home and abroad. Concerning the change of void ratio, Han et al.^10,11^ studied the axial compression performance of reinforced concrete hollow columns and found that the bearing capacity, ductility and deformation capacity of reinforced concrete hollow columns were poor. Al-Gasham et al.^12^ studied the axial compression performance of self-compaction concrete hollow columns with hollow ratio of 0.0%, 2.3%, 9.0% and 20.3%, and found that the ultimate load, stiffness and toughness of self-compaction concrete hollow columns were lower than those of solid columns, while the ductility was higher than that of solid columns. Concerning the change of section form of reinforced concrete column, Liang et al.^13,14^ focused on the research and investigation of the restraint effect of reinforced concrete solid columns with circular and square sections, reinforced concrete hollow columns with outer circle and inner circle sections, outer square and inner square sections under axial compression load. It was found that the restraint effect of reinforcement in solid columns and hollow columns on concrete deformation was different to a great extent, and the restraint effect of reinforcement in outer circle and inner circle, outer square and inner square concrete hollow columns was also different, and the difference was caused by the change of concrete expansion and confining pressure distribution on the cross section. To sum up, the following conclusions may be obtained that the own weight of the structure can be reduced and the ductility of the structure can be increased effectively via the increase of void ratio, while the bearing capacity is reduced, and the restraint effect of the reinforcement on concrete is different due to different opening methods.

Moreover, it can be found via the research that the composite column of angle steel concrete is featured with the advantages of high bearing capacity, good ductility and convenient construction^15^ when compared with the reinforced concrete columns at various section types, which can improve the axial compression performance of hollow columns of reinforced concrete. Hwang et al.^16^ studied the axial compression performance of the built-in angle steel composite column and found that the axial bearing capacity and deformation capacity of the test column were good, and sufficient lateral constraints were formed on the concrete in the core area via the angle steel and welded stirrups. Kim et al.^17^ studied the bearing capacity of built-in angle steel and high-strength concrete column and found that when the contribution and restraint efficiency of steel were high, the built-in angle steel and high-strength concrete column was still equipped with a large bearing capacity after the protective layer was peeled off because the strength of concrete in the core area remained unchanged after the angle steel yielded. On the basis, the paper aims to achieve a higher ultimate bearing capacity, deformation performance and better ductility of composite hollow column of steel fiber, high-strength lightweight aggregate concrete and angle steel with the purpose of reducing the own weight of the structure and improving the performance of reinforced concrete hollow column via replacing the ordinary concrete with steel fiber and high-strength lightweight concrete, and substituting the longitudinal reinforcement and stirrup with angle steel and batten plate respectively.

## Experimental study

### Test materials and their properties

#### Cementitious material

##### Cement

P·O 42.5 cement. The applicability can be seen in Table 1.

**Table 1:** Cement properties.

Specific surface area (m^2^/kg)	Setting time (min)		Compressive strength (MPa)		Flexural strength (MPa)	
	Initial setting	Final coagulation	3 days	28 days	3 days	28 days
360	120	180	26.6	54.8	5.2	8.3

##### Fly ash

Grade-I fly ash, loss on ignition is 0.6%, water content is 0.52%, water demand ratio is 95%.

##### Silica fume

Micro silica powder, loss on ignition is 3.7%, SiO_2_ content is 97.1%, water content is 0.5%, water demand ratio is 120%.

#### Aggregate

##### Fine aggregate

River sand, the particle grading is zone II medium sand, and the fineness modulus is 2.7.

##### Coarse aggregate

**Figure 1:**
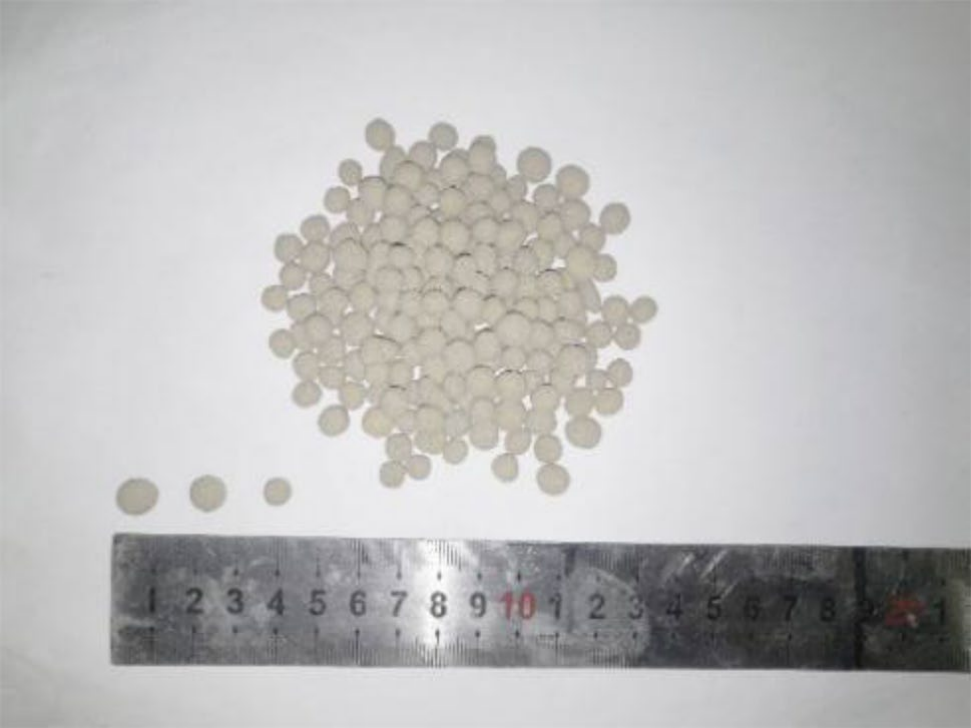
Coarse aggregate.

Crushed Stone shale ceramsite, particle size is 5–10 mm, bulk density is 1015 kg/m^3^, cylinder pressure strength is 17.7 MPa and 1 h water absorption is 8.1%, as shown in Fig. 1.

#### Steel

##### Steel

Q235 grade angle steel and flat steel. The material properties of the steel are tested according to ASTM E-8 standard. The performance parameters can be seen in Table 2.

**Table 2:** Steel properties.

Name	Yield strength (MPa)	Yield strain(× 10^–6^)	Tensile strength (MPa)	Modulus of elasticity (MPa)
Angle steel	327.6	2037	484.8	2.07 × 10^5^
Flat steel	298.6	1551	370.8	2.09 × 10^5^

#### Other materials

##### Steel fiber

**Figure 2:**
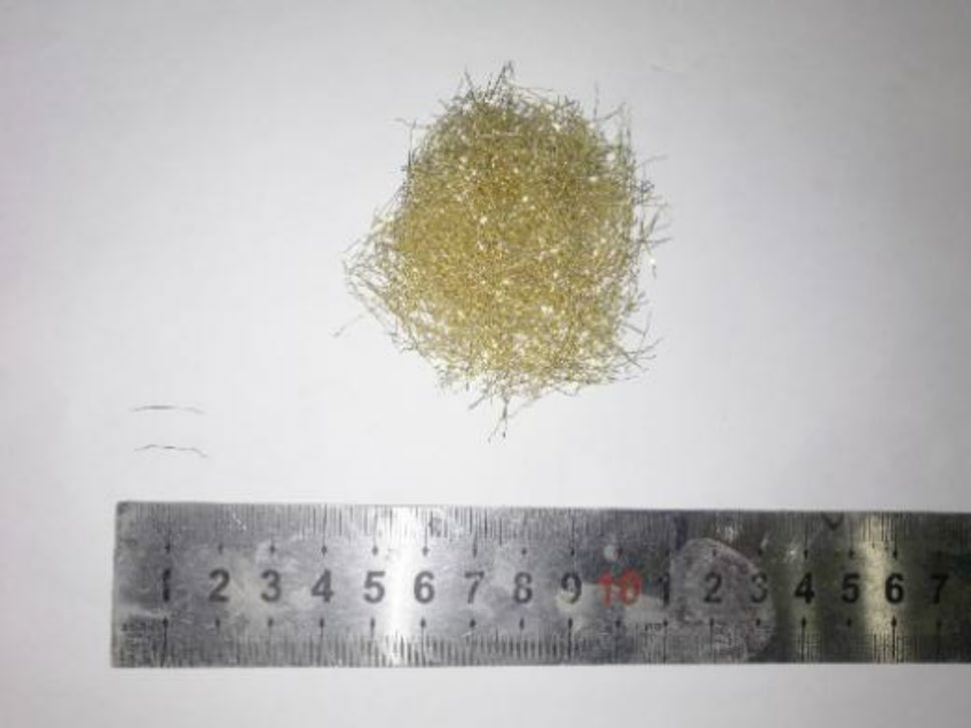
Steel fiber.

End hook type steel fiber, length is 13 mm, tensile strength is 1070 MPa and elastic modulus is 2 × 10^5^ MPa, as shown in Fig. 2.

##### Water reducing agent

Naphthalene B2 high-efficiency water reducing agent, water reducing rate is 19%.

### Concrete mix ratio

It is found that high-strength lightweight aggregate concrete has good structural benefits^18^, so it is widely used in practical projects, while there are a few relevant studies on the application of steel reinforced concrete columns^19^. Therefore, the high-strength lightweight aggregate concrete with strength grade of LC60 is used for the test in the paper, and the mix ratio can be shown in Table 3. The axial compression bearing capacity of the specimen is influenced slightly via adding the steel fiber into high-strength lightweight aggregate concrete with a three-dimensional random distribution^20^, which can effectively improve the overall performance of the test column and reduce the spalling degree of the concrete protective layer. Considering the workability of concrete, the steel fiber at 39 kg/m^3^ is added into the high-strength lightweight concrete in the paper.

**Table 3:** Mix proportion of concrete/(kg/m^3^).

Water	Cement	Sand	Ceramsite	Steel fiber	Water reducer
186	371	612	612	39	15

### Basis for selection of void ratio

The research results of Ji et al.^21^ show that the hollow ratio influences the mechanical performance of hollow columns greatly. In practical application, controlling the hollow ratio within 36% can ensure the safety of members. The research results of Al-Gasham et al.^22^ show that the void ratio of outer square and inner circle self compacting concrete hollow columns increases from 0 to 25.8%, the strength and stiffness of the test columns decrease, while the ductility increases slightly. The research results of Al-Ajarmeh et al.^23–25^ show that the strength and ductility of GFRP reinforced concrete hollow columns are higher than those of reinforced concrete hollow columns. When appropriate parameter design is adopted, the bearing capacity of GFRP reinforced concrete hollow columns is higher than that of GFRP reinforced concrete solid columns. Considering that the smaller hollow ratio has slight effect on the weight reduction of hollow columns, the construction difficulty is increased, the bearing capacity and ductility of hollow columns are reduced greatly at the larger hollow ratio, the hollow ratio in this test is taken as 0, 15%, 16%, 32% and 36%.

### Specimen design

**Figure 3:**
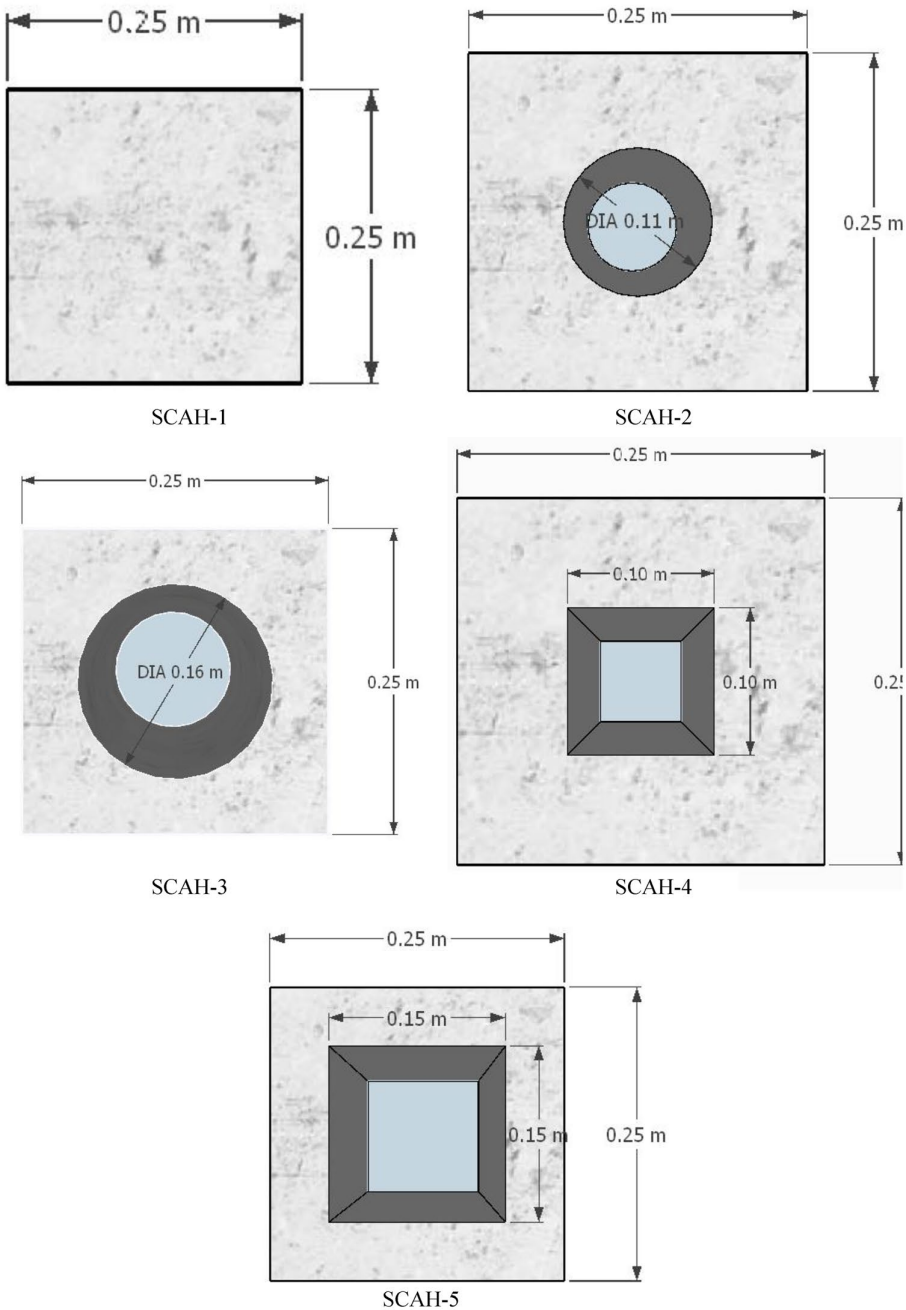
Cross section of the test column (Sketch up 2019 https://www.sketchup.com/).

Five SCAH column specimens have been made. The cross-sectional dimension of the column is 250 × 250 mm, the hollow diameter of round hole is 110 mm and 160 mm respectively, the hollow side length of square hole is 100 mm and 150 mm respectively, and the thickness of protective layer is 20 mm. The angle steel adopts Q235 steel with side length of 30 mm and thickness of 4 mm; The batten is made of Q235 flat steel with the thickness of 6 mm, the spacing of 150 mm and the height of 750 mm. The cross section is shown in Fig. 3, and the specific parameters are shown in Table 4. (In the sample number, S represents steel fiber, C represents concrete, A represents angle steel concrete column, and the figure followed is the number).

**Table 4:** Specimen variable parameters.

Specimen name	Hollow ratio (%)	Concrete strength (MPa)	Volume content of steel fiber (%)	Opening form
SCAH-1	0	LC60	0.5	Circular
SCAH-2	15	LC60	0.5	Circular
SCAH-3	32	LC60	0.5	Circular
SCAH-4	16	LC60	0.5	Square
SCAH-5	36	LC60	0.5	Square

### Loading device and loading scheme

**Figure 4:**
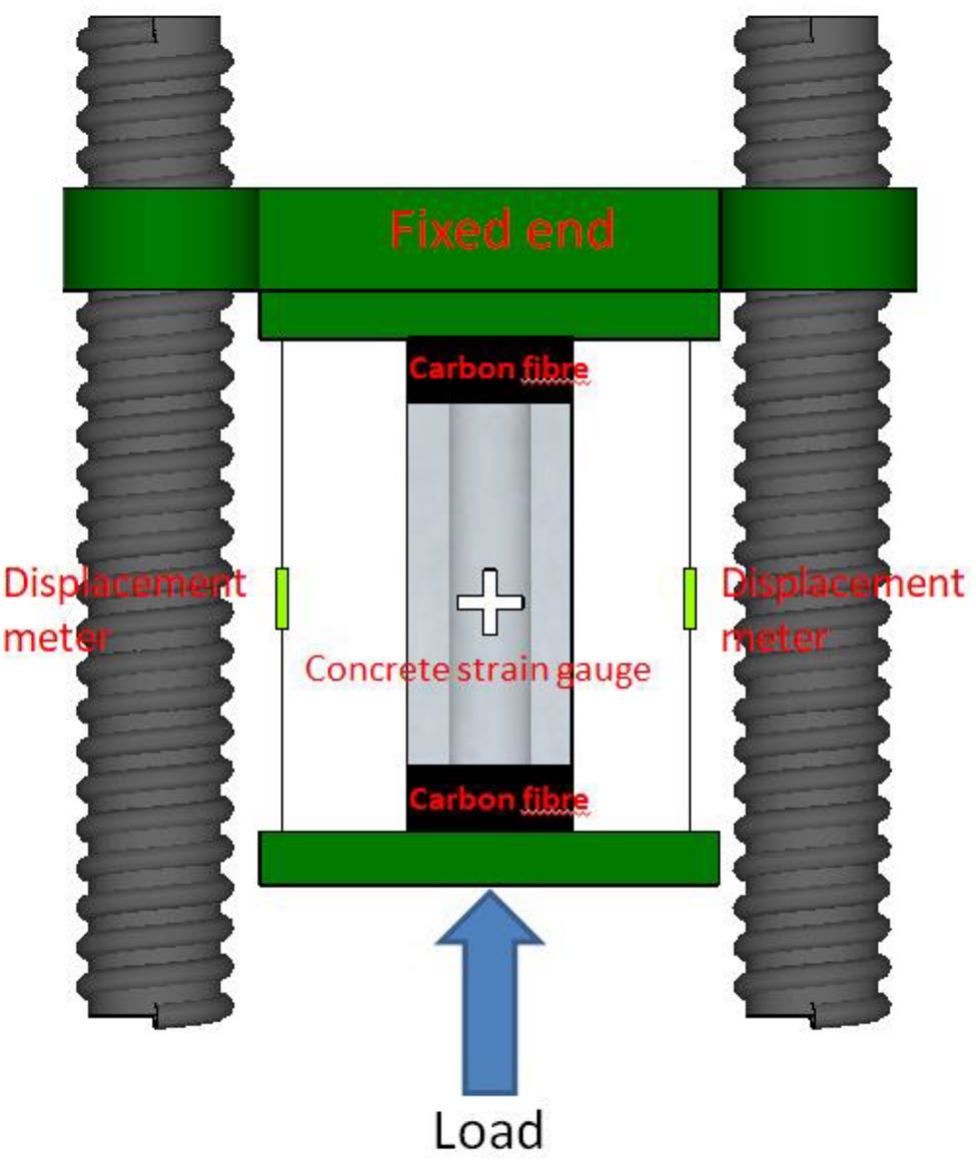
Loading device (Sketch up 2019 https://www.sketchup.com/).

**Figure 5:**
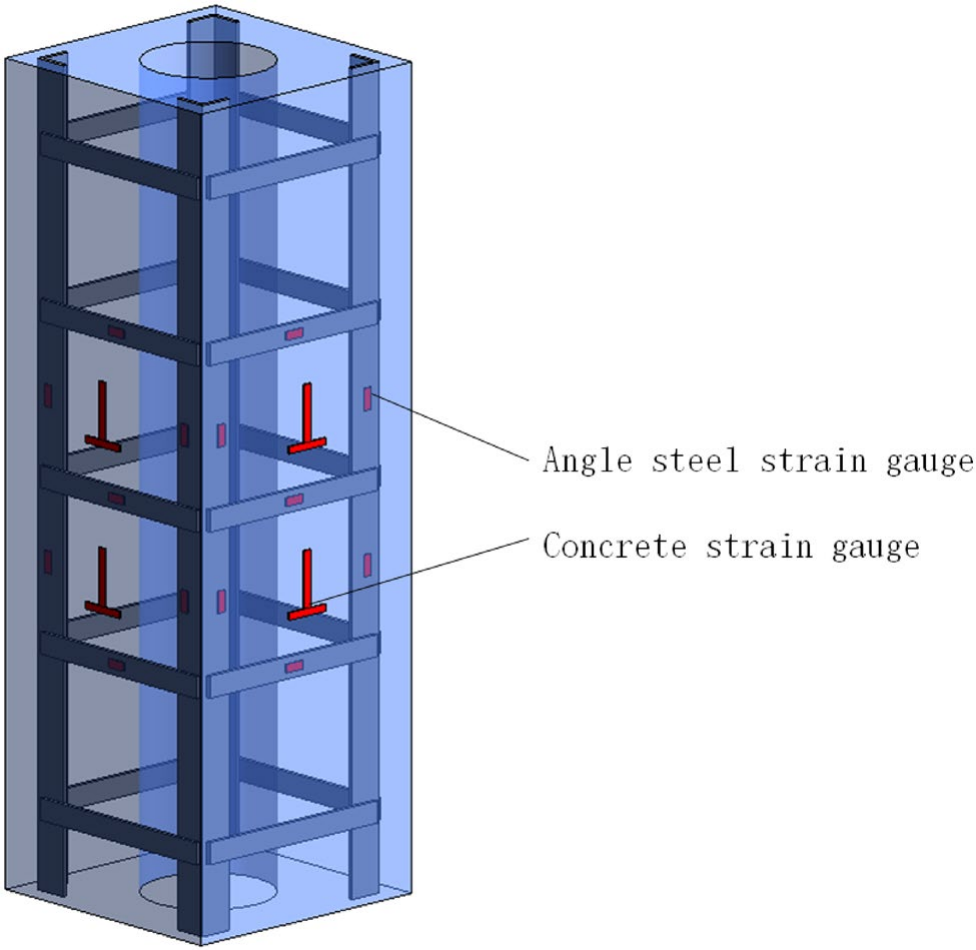
Layout of the steel strain gauges (Rivet 2018 http://www.chinarevit.com/).

5000 kN hydraulic testing machine is used for loading test. According to *Concrete Structure Test Method Standard* (GB/T50152-2012^26^), the method of force control to load step by step is used in the test. At the beginning of loading, the load of each level is 1/10 of the estimated *P*_*u*_ (*P*_*u*_ represents the peak load) and the load is maintained for 2 min; When the load reaches the estimated 0.8 *P*_*u*_, the load of each level is 1/20 of the estimated *P*_*u*_, and the load is held for 2 min; When the load drop section is close to 0.6*P*_*u*_, It is loaded continuously and slowly until the specimen is finally damaged and the loading is stopped. Figure 4 shows the arrangement of loading device, displacement meter and concrete strain gauge on the surface of the test piece, and Fig. 5 shows the arrangement of angle steel and batten plate strain gauge.

## Test results and discussion

### Failure mode of specimen

**Figure 6:**
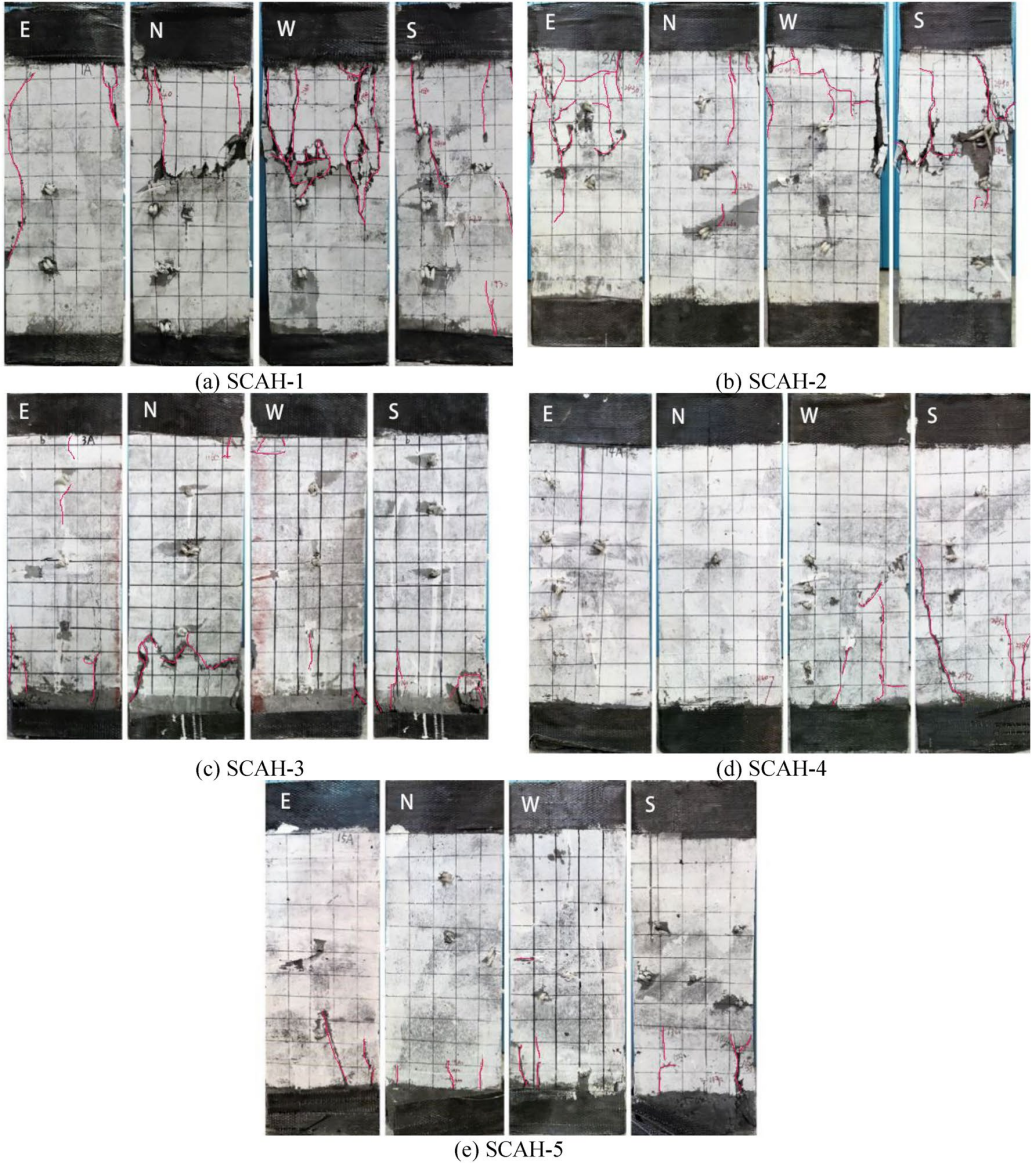
Typical failure modes of specimens (Adobe Photoshop 2020 https://www.adobe.com/products/photoshop.html).

At the initial stage of loading, the specimen lies in the elastic stage, the concrete is compressed longitudinally without obvious crack on its outer surface. When the load reaches about 0.3*P*_*u*_–0.6 *P*_*u*_ of the peak load, some narrow and short vertical cracks begin to appear at the end of the cylinder; With the gradual increase of load strength, the number of longitudinal cracks is also increasing, while the cracks extend slowly to the middle of the specimen and the width and depth of cracks increase gradually under the restraint and obstruction of batten plate; The load reaches about 0.6 *P*_*u*_− 0.8 *P*_*u*_ of the peak load, and the crackling sound and skin blasting phenomenon appear successively at the surface of the column; After the load reaches the peak load, the through longitudinal splitting cracks and transverse cracks emerge rapidly on the column surface at a speed visible to the naked eye; the loading is continued. When the load is reduced to 0.6 *P*_*u*_ of the peak load, the angle steel of all specimens buckles obviously, some batten plates bulge out, and the concrete is crushed and damaged in different degrees. At the corner of the bottom of the column, some concretes are crushed and peeled off, and the loading is stopped. The typical failure mode of the specimens is shown in Fig. 6, the following may be obtained from the figure: (1) It is found that the concrete of round hole specimens at 1/3 or 2/3 of the column is seriously damaged with large transverse crack width and the separation of angle steel and the concrete via comparing the failure phenomena of round hole and square hole specimens, there is no peeling due to the bridging effect of internal steel fiber, and the failure phenomenon inside the round hole is not obvious; The specimen with square hole has obvious inclined longitudinal splitting cracks at the bottom of the column, part of the concrete at the bottom is crushed and peeled off, and the damage trace of the bottom and corner inside the square hole is obvious. The reason for this phenomenon is that the arch effect is formed on the inner wall of the circular hole, which makes the overall stress on the wall of the circular hole more uniform and supports the concrete. The stress concentration at the edges and corners of the square hole column is obvious, which is caused by the uneven stress on the inner wall of the square hole column.

With the increase of the hollow ratio coefficient of the hollow column, the damage degree of the outer surface of the hollow column with circular hole is weakened, and the damaged section moves down from 2/3 to 1/3. It is because the wall thickness gradually decreases along with the increase of the hollow ratio. In the later stage of loading, the stress distribution of the section is uneven, and the transverse strain in the middle of the four sides of the hollow column is gradually greater than the corner strain. The transverse expansion of concrete becomes more and more obvious with the increase of the load, the non-uniformity of the whole force of the hollow column is amplified, resulting in the destruction of the section near the loading end; The opening form is a hollow column with square holes, and the damage phenomenon is basically the same as the damage degree.

The constraint at the end of the hollow column is very small, so the damage at the end of the concrete is relatively serious. According to the stress nephogram simulated by finite element, the stress at the end of the concrete is more concentrated, it can be seen that the number and width of cracks at the end of the sample increase, and continue to extend to the middle of the sample. However, the end restraint of solid concrete column is well, and the specimens are mainly destroyed in the middle.

### Load-longitudinal deformation curve

**Figure 7:**
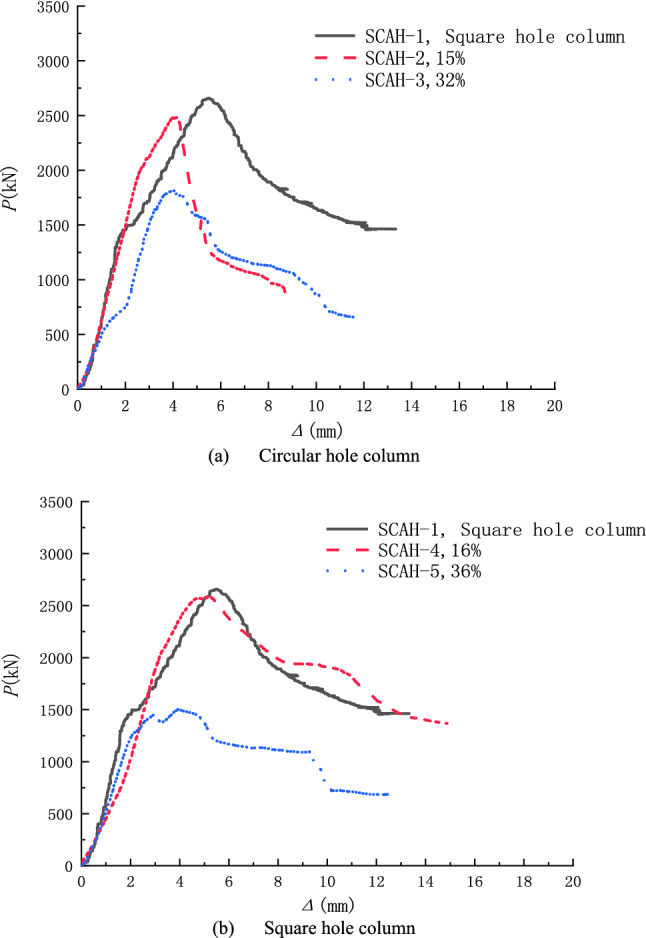
Axial load-longitudinal deformation curves of all specimens (origin 2019b https://www.originlab.com/).

The load-deformation curve of the specimen is shown in Fig. 7. It can be seen from the figure that the linear relationship is shown between them at the initial stage of load action, and the deformation of the specimen increases with the increase of load. At this time, the specimen is in the elastic stage. With the continuous increase of load, the slope of the load deformation curve of the specimen begins to decrease, and the increase rate of deformation is gradually greater than that of load. At this moment, plastic deformation occurs in concrete and steel in different degrees until the peak load is reached. Compared with 5 (a) and 5 (b) in the above figure, it can be seen that the yield and peak load of the specimen are significantly reduced along with the increase of void ratio; For the specimens with small void ratio (such as SCAH-2 and SCAH-4), the hollow column with round hole and square hole is similar to the solid column at the rising section of the curve, and the peak load of the hollow column with square hole is closer to the solid column; In the descending section of the curve, the specimens of hollow columns with square holes are similar to those of solid columns. The descending section of the curve is gentle and the bearing capacity decreases slowly, and the favorable ductility is shown. The ductility of hollow columns with square holes is slightly better than that of solid columns; The falling section of the curve of the round hole specimen is steep, the bearing capacity decreases rapidly, and the poor ductility is shown. For the specimens with large void ratio (such as SCAH-3 and SCAH-5), the yield and peak load of the specimens are significantly reduced. The peak load of the specimens with round holes is higher than that of the specimens with square holes, and the ultimate deformation performance of the specimens with square holes is significantly better than that of the specimens with round holes. The comparison of curves shows that the ability of resisting plastic deformation of hollow columns with square holes is better than that of hollow columns with circular holes; Under the condition of small void ratio, even the yield load of SCAH-4 concrete hollow column is higher than that of SCAH-1 concrete solid column, indicating that the small void ratio will not have too much adverse impact on the axial compression bearing capacity of hollow column. The test results are shown in Table 5.

**Table 5:** Test results.

Specimen name	Yield load/kN	Yield deformation/mm	Peak load/kN	Peak deformation/mm	μ
SCAH-1	2393.2	4.55	2659.0	6.34	1.49
SCAH-2	2325.1	3.54	2480.7	4.09	1.28
SCAH-3	1806.3	3.92	1814.0	3.91	1.38
SCAH-4	2544.8	4.54	2590.2	5.35	1.51
SCAH-5	1433.7	2.80	1502.7	3.96	1.86

### Strain analysis

#### Longitudinal strain analysis of angle steel

**Figure 8:**
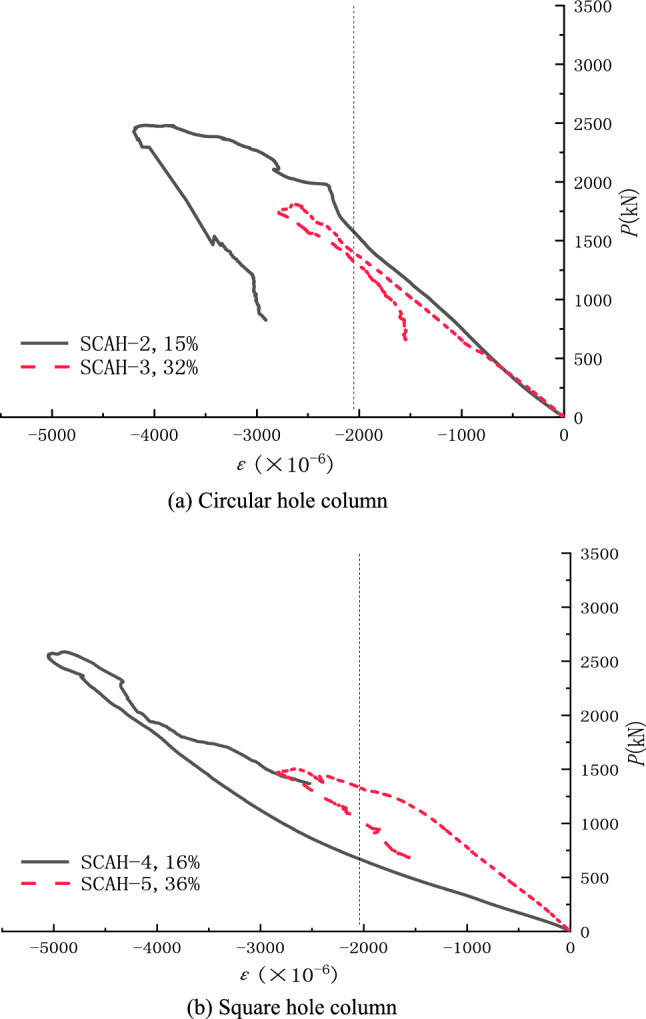
Typical axial load-strain curves (origin 2019b https://www.originlab.com/).

Load-angle steel longitudinal strain (P-ε) curve of the specimen can be shown in Fig. 8. It can be seen from the figure that the angle steel of each specimen has reached yield (yield strain) at the rising section of load ε Y is 2.037 × 10^–3^). Before the longitudinal angle steel reaches the yield strain, the strain of the longitudinal angle steel gradually increases with the increase of void ratio under the same load. Due to the obvious corner effect of the inner wall of the hollow column with square hole, the longitudinal stiffness of the high-strength lightweight aggregate concrete hollow column is weakened, so that the longitudinal angle steel strain of the square hole hollow column is greater than that of the round hole hollow column with the same hollow rate.

#### Concrete strain analysis

**Figure 9:**
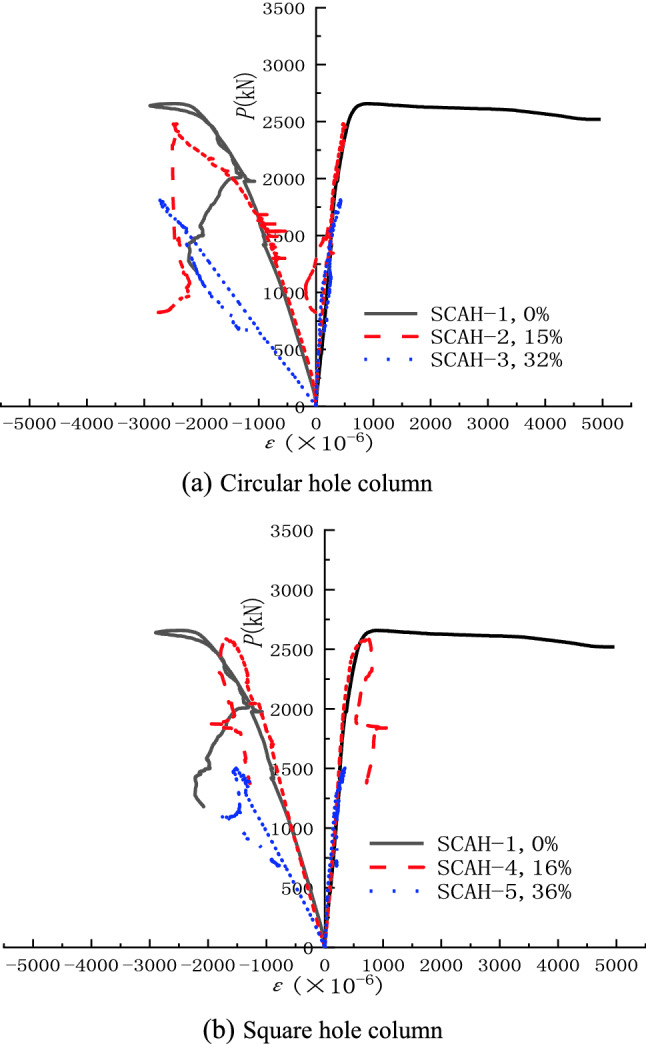
Compressive stress-surface strain curves of concrete (origin 2019b https://www.originlab.com/).

The load-concrete transverse and longitudinal strain curves of the specimen (the left side of the coordinate is the longitudinal strain of the concrete and the right side is the transverse strain of the concrete) can be shown in Fig. 9. It can be seen from the figure that the transverse strain development of solid column is more sufficient than that of hollow column, and the form of opening has little effect on the strain development of specimen; Before the peak load is reached, the transverse strain development of hollow columns under different void ratios is basically the same, while the longitudinal strain is quite different; Under the same axial load, the longitudinal strain of hollow column is significantly greater than the transverse strain; Under the condition of the same void ratio, the longitudinal limit strain of hollow column with square hole is quite different from that of solid column, while the longitudinal limit strain of hollow column with circular hole is slightly lower than that of solid column, indicating that circular hole hollow column is better utilized in material.

### Ductility

**Figure 10:**
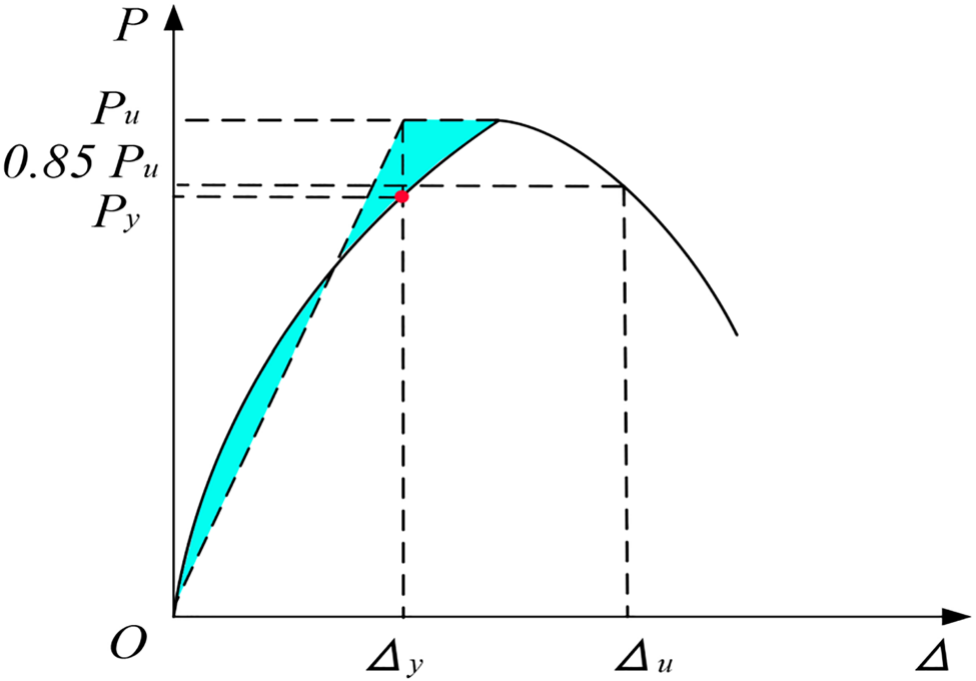
Equvialent elasto-plastic energy method (Visio 2019 https://www.microsoftstore.com.cn/software/office/visio-standard-2021).

The ratio of ultimate deformation and yield deformation is defined as the deformation ductility coefficient, the deformation corresponding to 0.85pu in the descending section of the load-deformation curve is taken as the ultimate deformation, and the yield deformation of the specimen is determined by the equal energy method^27^, as shown in Fig. 10, it can be seen from Table 5: the ductility coefficient increases gradually along with the increase of void ratio. Under the same void ratio, the ductility coefficient of hollow column with square hole is slightly higher than that of hollow column with round hole. On the whole, the brittleness of the specimen is relatively large and it is prone to have the brittle failure; By comparing the ductility of solid column and hollow column, it can be seen that the ductility coefficient of hollow column with square hole is higher than that of solid column. It is because the concrete of hollow column expands to the internal gap under the action of axial pressure, so the longitudinal deformation of hollow column is larger than that of solid column under the same stress. It can be seen that appropriate void ratio is beneficial to improve the ability of hollow column to resist plastic deformation.

## Numerical simulation of axial compression performance of composite hollow column of steel fiber, high-strength lightweight aggregate concrete and angle steel

### Material constitutive relation

#### Constitutive model of concrete

**Figure 11:**
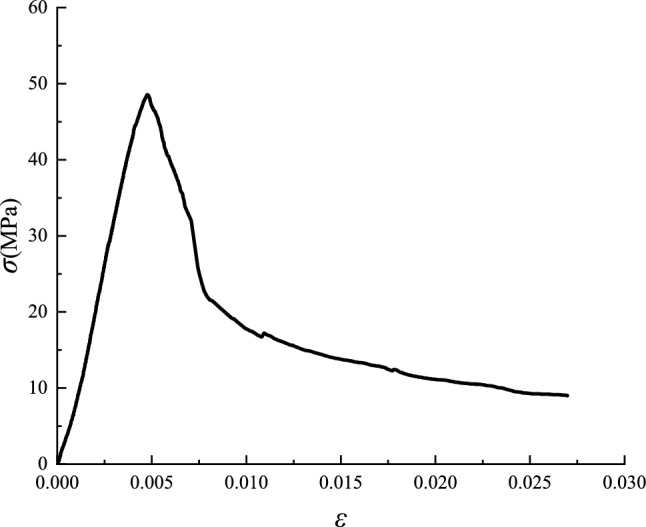
Stress–strain curve (origin 2019b https://www.originlab.com/).

The high-strength lightweight concrete is obtained from the full stress–strain curve test (as shown in Fig. 11) on the prismoid specimen of 150 × 150 × 300 mm, and it is simulated with concrete plastic damage model.

#### Constitutive model of steel

**Figure 12:**
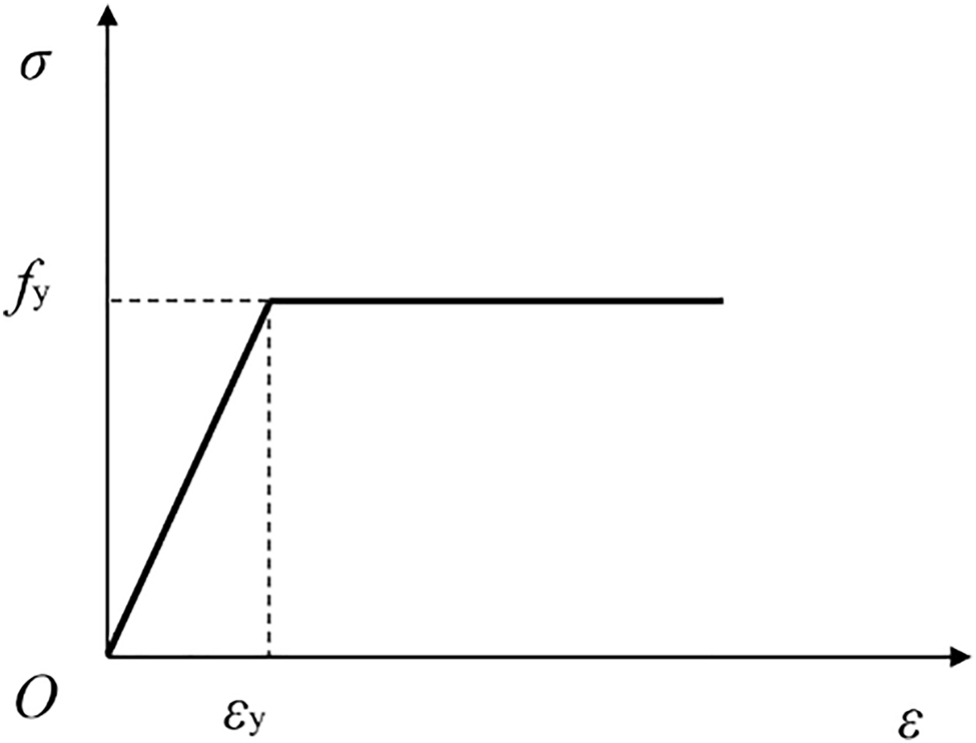
Ideal elastoplastic model (Microsoft PowerPoint 2019 https://www.microsoft.com/zh-cn/microsoft-365/powerpoint).

The stress–strain relationship of steel adopts the ideal elastic–plastic model (Fig. 12) provided in ABAQUS and meets the von Mises yield criterion, as shown in Eqs. (1) and (2).

1 $$ {\text{When}}\;\varepsilon_{y} \le \varepsilon_{s} ,\;\sigma_{s} = f_{y} . $$

2 $$ {\text{When}}\;\varepsilon_{y} \le \varepsilon_{s} ,\;\sigma_{s} = f_{y} . $$

In the formula: $\sigma_{s}$, $\varepsilon_{s}$ and $E_{s}$ represent the stress, strain and elastic modulus of steel respectively; $f_{y}$ represents the yield strength of steel; $\varepsilon_{y}$ represents the yield strain corresponding to yield strength.

### Finite element modeling process

**Figure 13:**
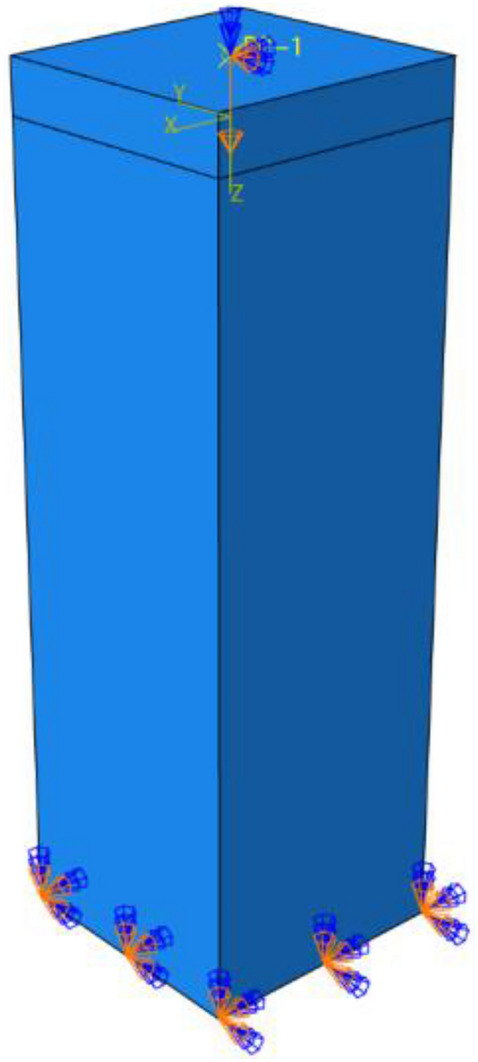
Loads and boundary conditions (ABAQUS 2016 https://www.3ds.com/products-services/simulia/).

The finite element analysis model of SCAH column is established by using ABAQUS software. High-strength lightweight concrete, steel base plate and angle steel skeleton are simulated by eight nodes reduced integral three-dimensional solid element C3D8R. Angle steel and batten plate shall be combined as the angle steel skeleton. The angle steel skeleton is built into the concrete, and the concrete and steel base plate are bound and connected. The top surface of the steel cushion block is coupled as the center point. The axial displacement loading control is used for the finite element simulation, and the vertical displacement is applied at the coupling point of the top surface of the steel cushion block. The concrete bottom is fixed completely as shown in Fig. 13.

**Figure 14:**
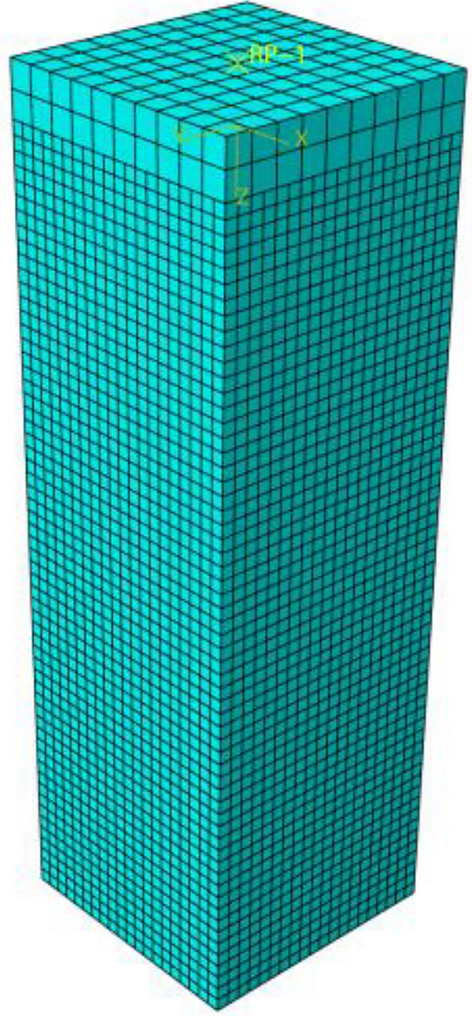
Grid division of concrete and base plate (ABAQUS 2016 https://www.3ds.com/products-services/simulia/).

**Figure 15:**
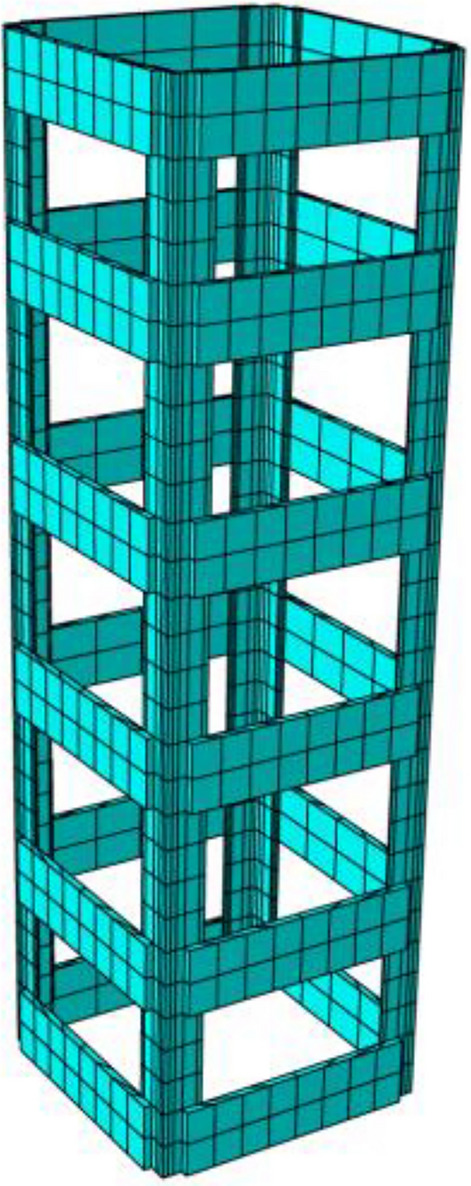
Mesh division of angle steel cage (ABAQUS 2016 https://www.3ds.com/products-services/simulia/).

Through the analysis of mesh sensitivity in the early stage of finite element simulation, it can be found that when the concrete mesh size is 12.5 mm, if the mesh size continues to be reduced, the calculation accuracy of finite element simulation is less affected, while the calculation time increases more. At the same time, taking the concrete grid as 12.5 can avoid using C3D8R element to simulate the hourglass mode of concrete, thus ensuring the specimen with the largest void ratio is divided into at least 4 elements along the thickness direction. The mesh size of angle steel cage and steel base plate is 25 mm. Before grid generation, the irregular parts should be divided into structured grids. The mesh division of concrete, base plate and angle steel framework can be seen in Figs. 14 and 15.

**Figure 16:**
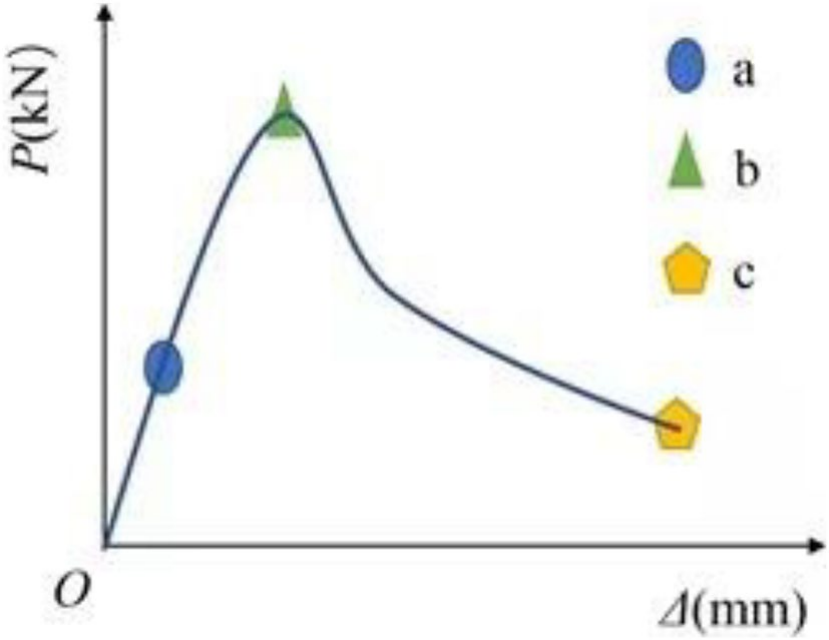
Typical load longitudinal deformation curve (Microsoft PowerPoint 2019 https://www.microsoft.com/zh-cn/microsoft-365/powerpoint).

The stress distribution of concrete and angle steel skeleton of the above five SCAH columns during the whole stress process is analyzed. In order to facilitate the analysis, the load-longitudinal deformation curve of the specimen is divided into three stages (see Fig. 16): elastic section (OA), where the linear relationship exists between the load and longitudinal deformation of the specimen; Elastic–plastic section (AB),where the plastic deformation occurs in the concrete and steel, the load growth of the specimen at the stage is less than that of longitudinal deformation, the curve is slightly convex, the slope decreases gradually, and the ultimate bearing capacity of the specimen is reached at point B; Descending section (BC), where the curve enters into the descending section after reaching the peak point.

### Model verification

**Figure 17:**
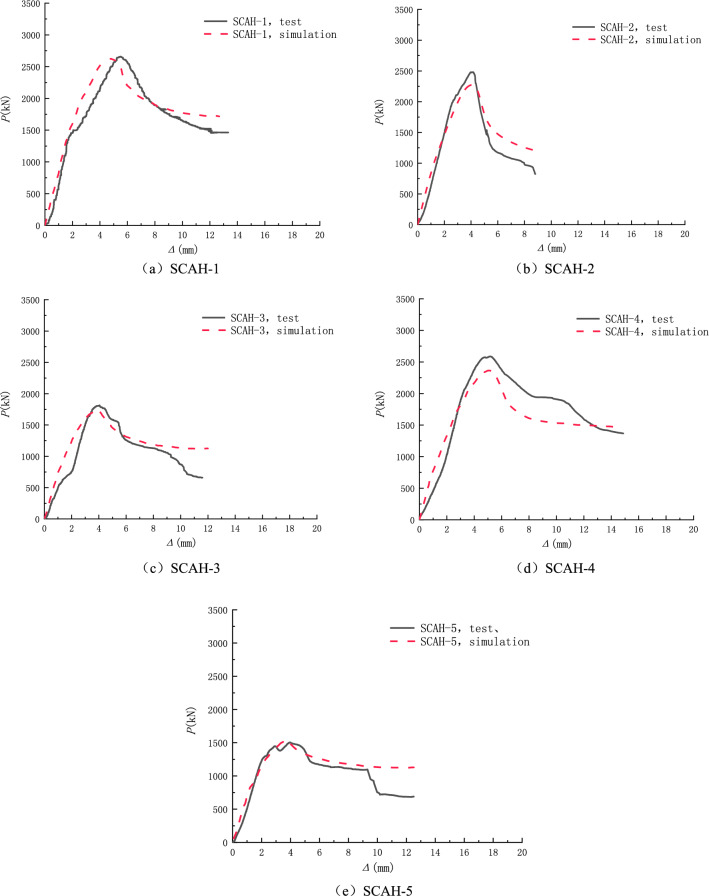
Finite element model verification (origin 2019b https://www.originlab.com/).

The finite element simulation is carried out on five specimens according to the above method, and the correctness of the finite element model is verified through the load-longitudinal deformation curve. It can be seen from Fig. 17 that the test results of the specimens are in good agreement with the finite element simulation results, and the error of the peak load is about 10%.

### Result analysis

#### Longitudinal stress distribution of concrete

**Figure 18:**
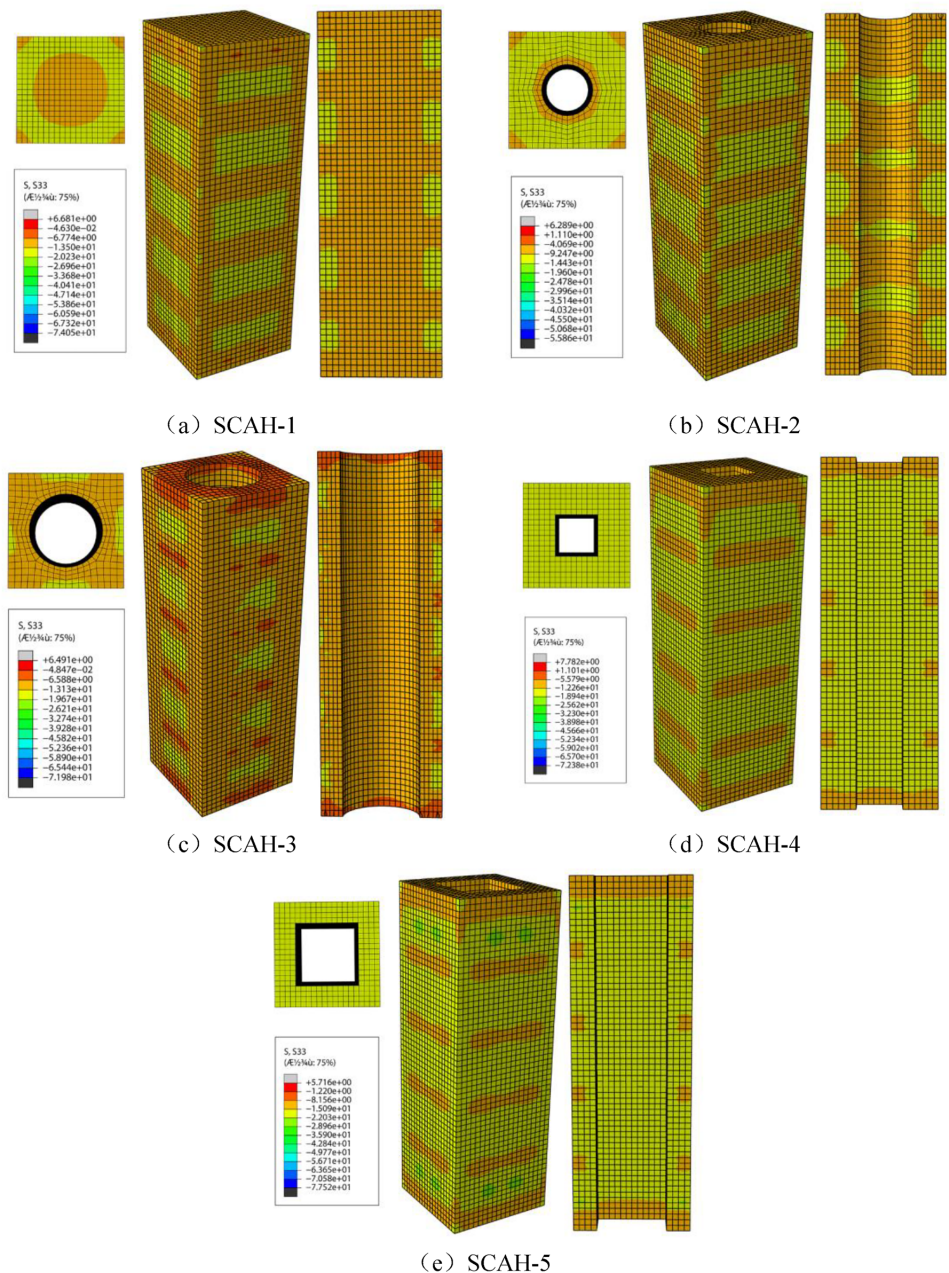
Cloud diagram of longitudinal stress of concrete at point A (ABAQUS 2016 https://www.3ds.com/products-services/simulia/).

The longitudinal stress (S33) represents the stress in the z-axis. The positive value is tensile stress and the negative value is compressive stress. It can be seen from Fig. 18 that the concrete is in the elastic stage at point A, the concrete compressive stress at the batten plate is less than that between the batten plates, in which the concrete compressive stress at the batten plate is about − 6 to − 13 MPa, the concrete compressive stress between the batten plates is about − 13 to − 20 MPa, and the concrete compressive stress is obviously less than its axial compressive strength.

**Figure 19:**
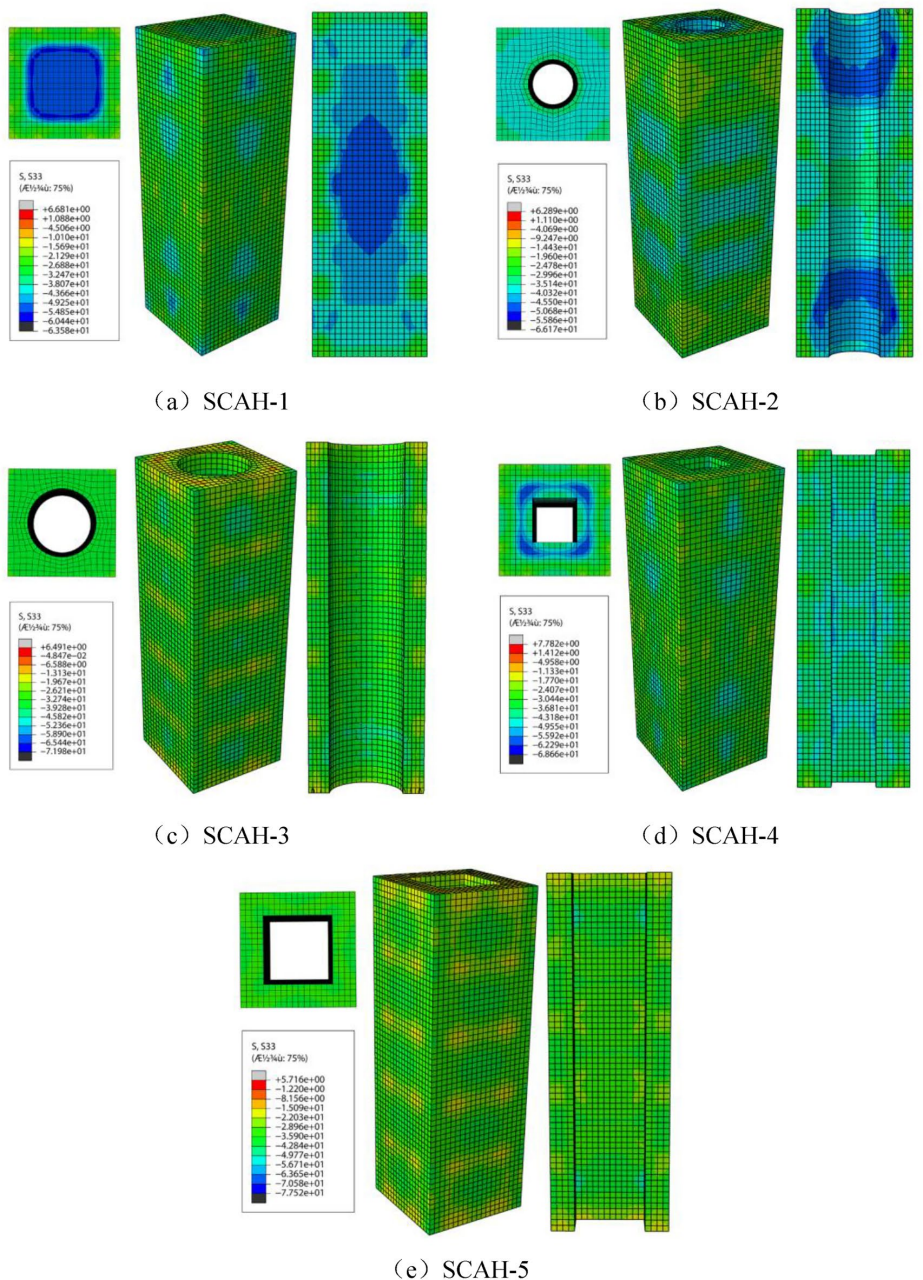
Cloud diagram of longitudinal stress of concrete at point B (ABAQUS 2016 https://www.3ds.com/products-services/simulia/).

It can be seen from Fig. 19 that the concrete exceeds the plastic stage along with the increase of the load between point A and point B, where the plasticity begins to develop and it lies in the elastic–plastic stage. The maximum compressive stress in the concrete core area is significantly increased for the specimen with small void ratio at point B, where the axial compressive strength reaches − 60 MPa with the increase of about 25%; For the specimens with large void ratio, the maximum compressive stress in the concrete core area increases slightly compared with that in point A, and the compressive stress of concrete increases to − 26 to − 36 MPa. The maximum compressive stress of SCAH-1 column is distributed near the core concrete of the middle section of the specimen, the maximum compressive stress of SCAH-2 column is distributed near the inner wall of the concrete at the height of the third point of the specimen (except the middle section), and the maximum compressive stress of SCAH-4 column is distributed near the corner of the concrete square hole at the height of the fourth point of the specimen.

**Figure 20:**
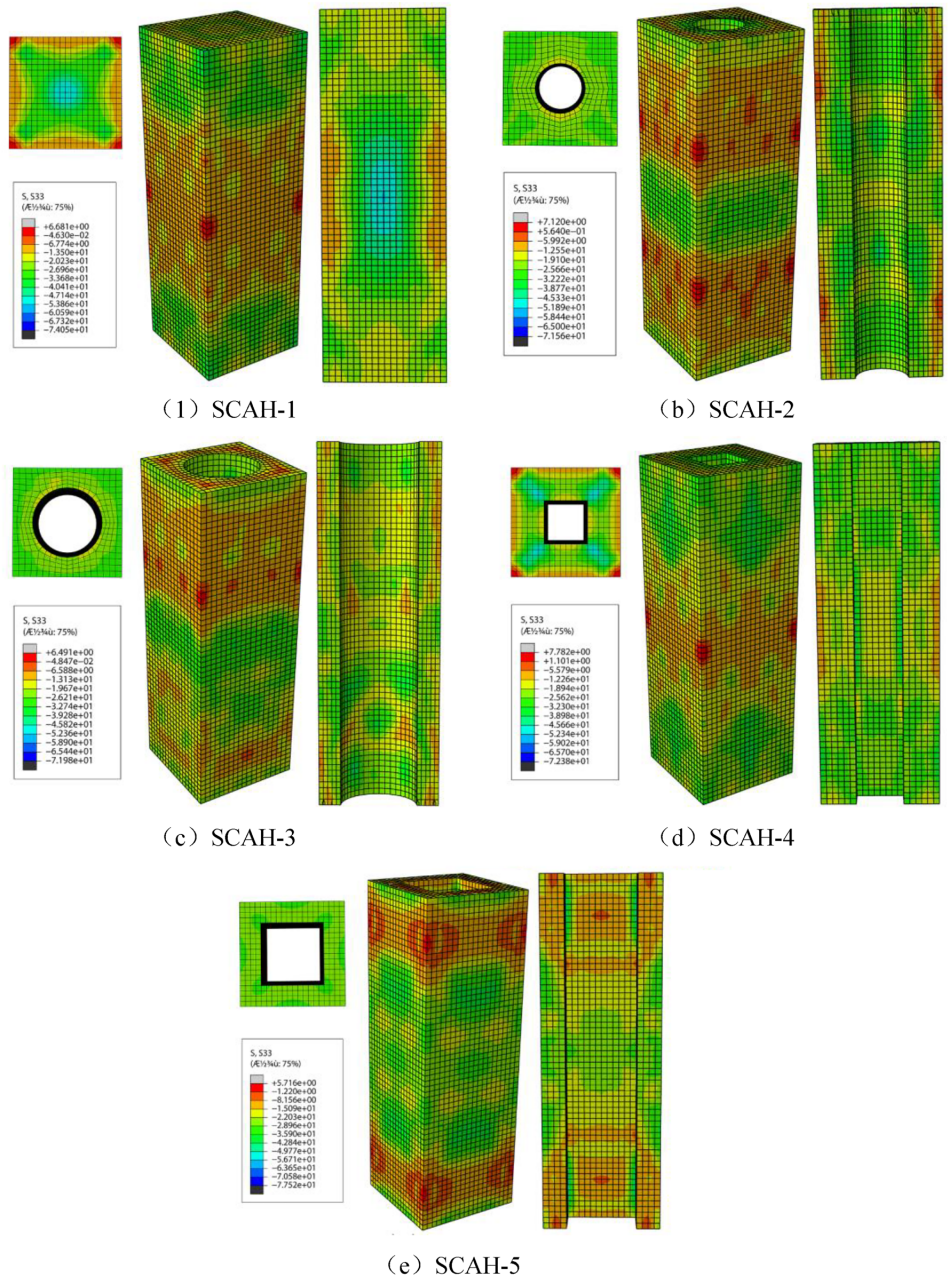
Cloud diagram of longitudinal stress of concrete at point C (ABAQUS 2016 https://www.3ds.com/products-services/simulia/).

It can be seen from Fig. 20 that there is a descending section between point B and point C. The compressive stress on the outer surface of the concrete of the specimen is small (about − 6 to − 13 MPa), and even small tensile stress (0–8 MPa) occurs along with the increase of vertical deformation. The maximum compressive stress of SCAH-1 column is distributed near the core concrete at the middle section of the specimen, and the compressive stress of the core concrete is the largest (about − 50 MPa). The compressive stress distribution of concrete near the circular hole in the middle section of hollow column with circular hole is relatively uniform with the value of about − 20 to − 30 MPa. The concrete compressive stress at the corner of the square hole is greater than that near the midpoint of the side of the square hole. In particular, the concrete stress concentration at the corner of the section in SCAH-4 specimen is obvious with the value of about − 50 MPa.

#### Stress distribution of angle steel skeleton

**Figure 21:**
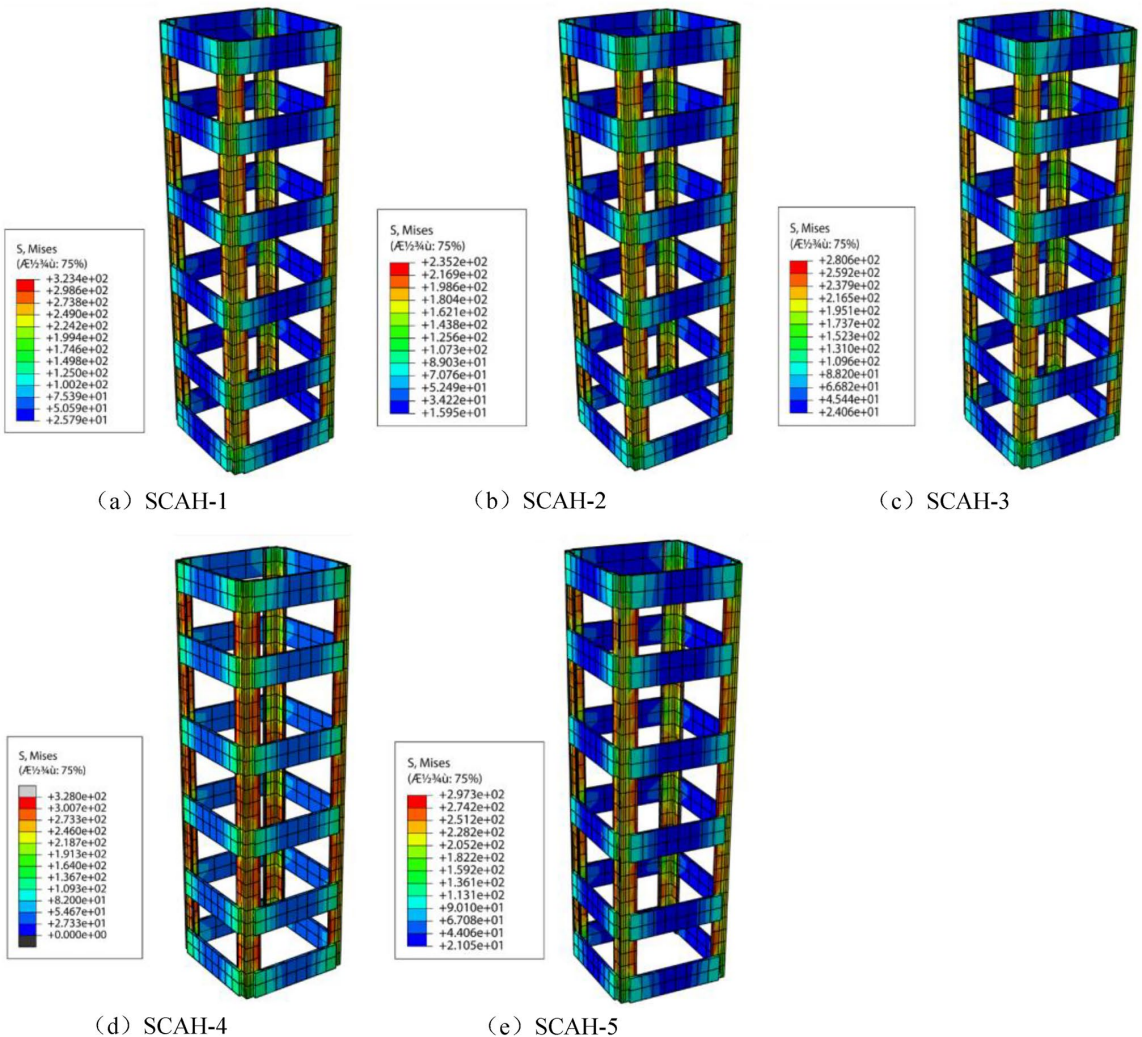
Mises cloud diagram of angle steel skeleton at point a (ABAQUS 2016 https://www.3ds.com/products-services/simulia/).

**Figure 22:**
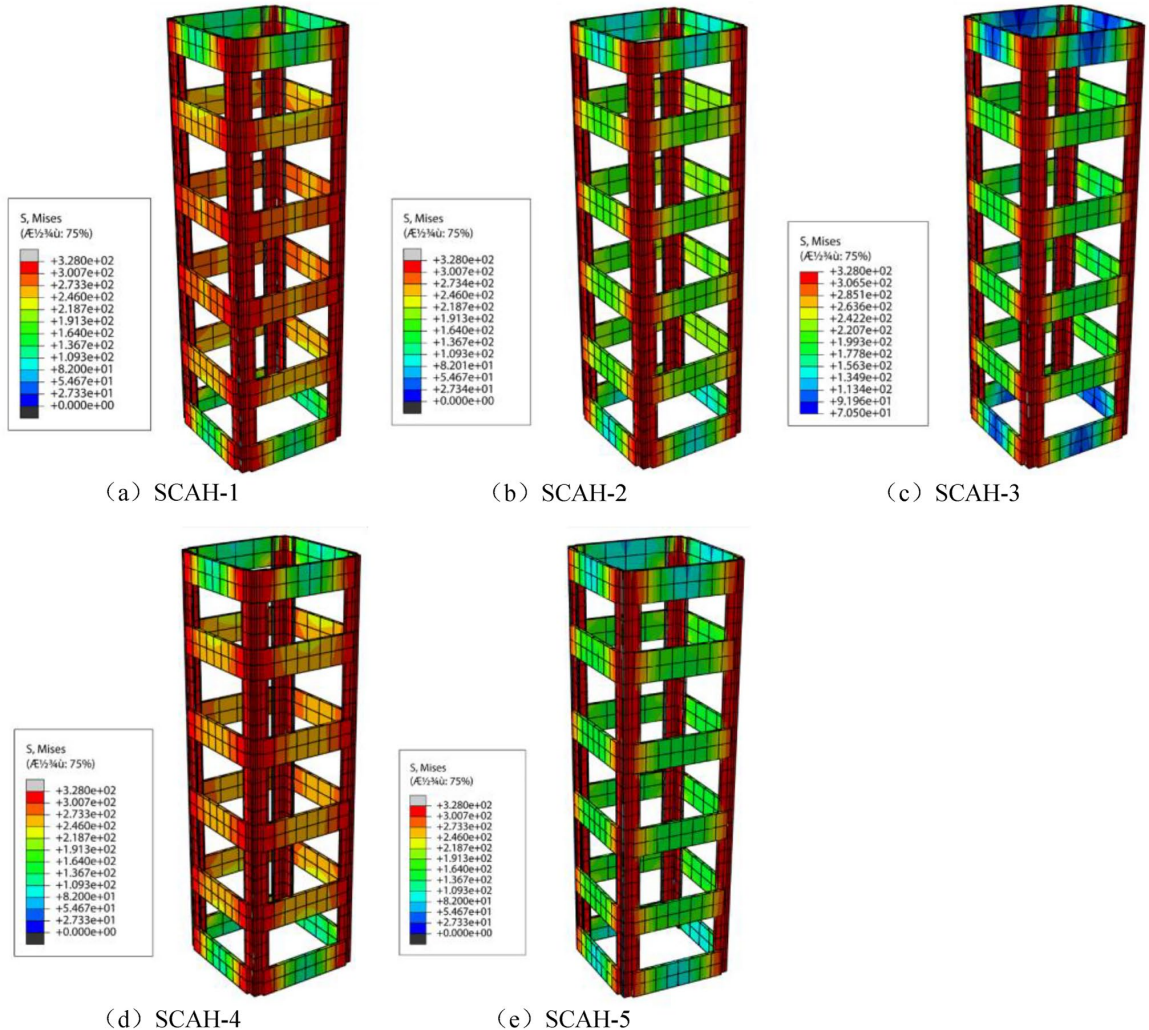
Mises cloud diagram of angle steel frame at point B. (ABAQUS 2016 https://www.3ds.com/products-services/simulia/).

**Figure 23:**
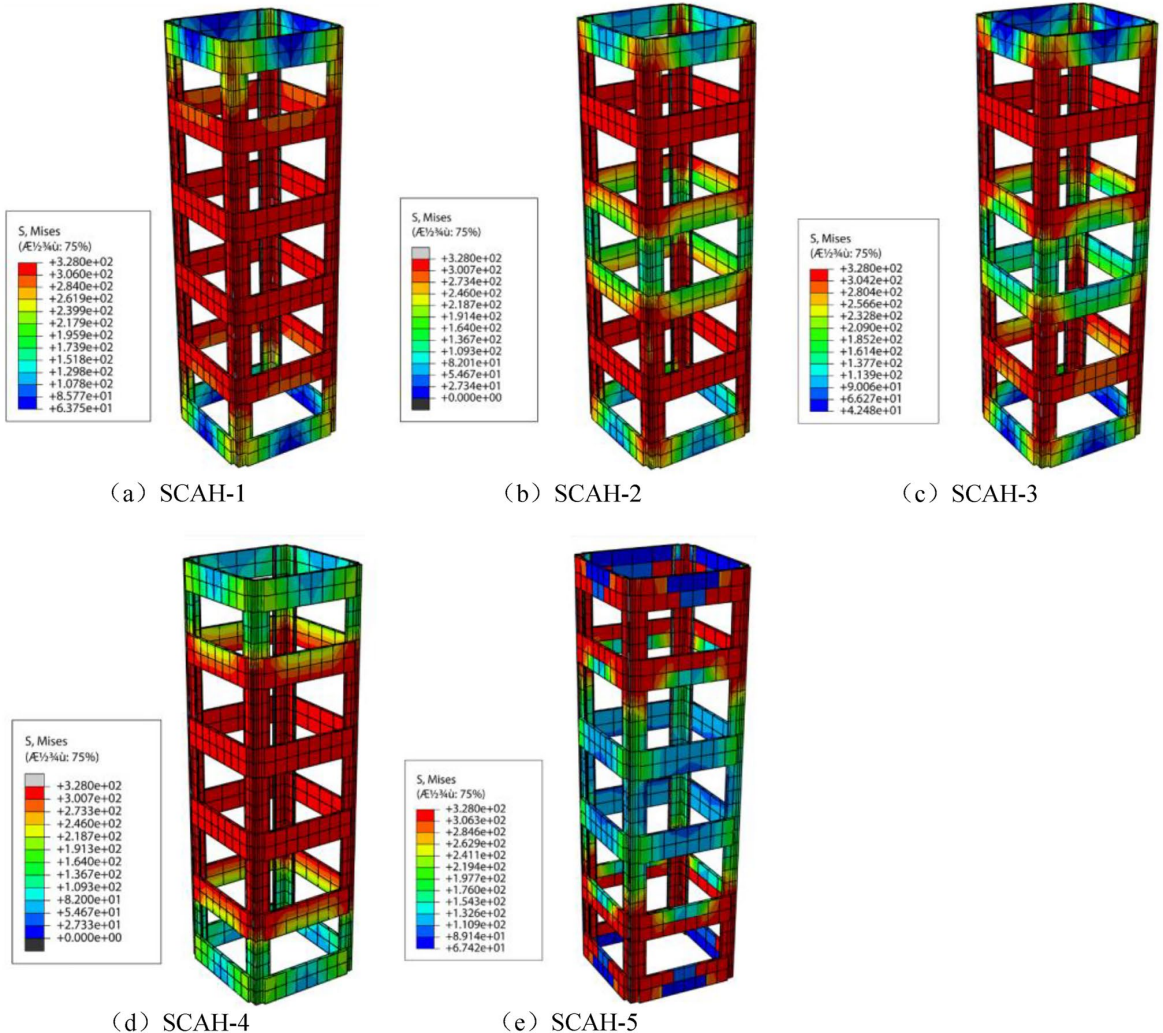
Mises cloud diagram of angle steel frame at point C (ABAQUS 2016 https://www.3ds.com/products-services/simulia/).

Von Mises stress is the fourth strength theory (such as Eq. (3)). According to the principle of energy conservation, it is used to judge whether the material yields. Similarly, the stress of angle steel skeleton is divided into three stages (as shown in Figs. 21, 22, 23): in the elastic stage (OA), the Mises stress of angle steel is significantly greater than that of batten plate, and the Mises stress of angle steel is less than 300 MPa, but it does not reach the yield stress; At the same time, the stress of the angle steel between the two battens is significantly greater than that at the batten, and the Mises stress near the batten near the angle steel is significantly greater than that near the center of the batten.

3 $$ (\delta_{{_{1} }} - \delta_{2} )^{2} + (\delta_{2} - \delta_{3} )^{2} + (\delta_{3} - \delta_{1} )^{2} = 2\delta_{s}^{2} . $$

The specimen enters into the elastic–plastic stage (AB) along with the increase of the load, and the yield stress (328 MPa) is reached within the full length of the angle steel, while the batten stress is about 160–300 MPa with the failure to reach the yield stress. At this time, the Mises stress of the middle batten of the specimen is significantly greater than that of the end batten, and the Mises stress of the batten at the connection with the angle steel is significantly greater than that near the center point of the batten.

The specimen enters into the descending section (BC) along with the development of the loading, the radial deformation of concrete increases rapidly, the tensile stress of batten plate increases, and finally it reaches the yield stress. Most of the specimens yield to the second and fifth battens.

## Simplified calculation of axial compression bearing capacity of composite hollow column of steel fiber, high-strength lightweight aggregate concrete and angle steel

**Figure 24:**
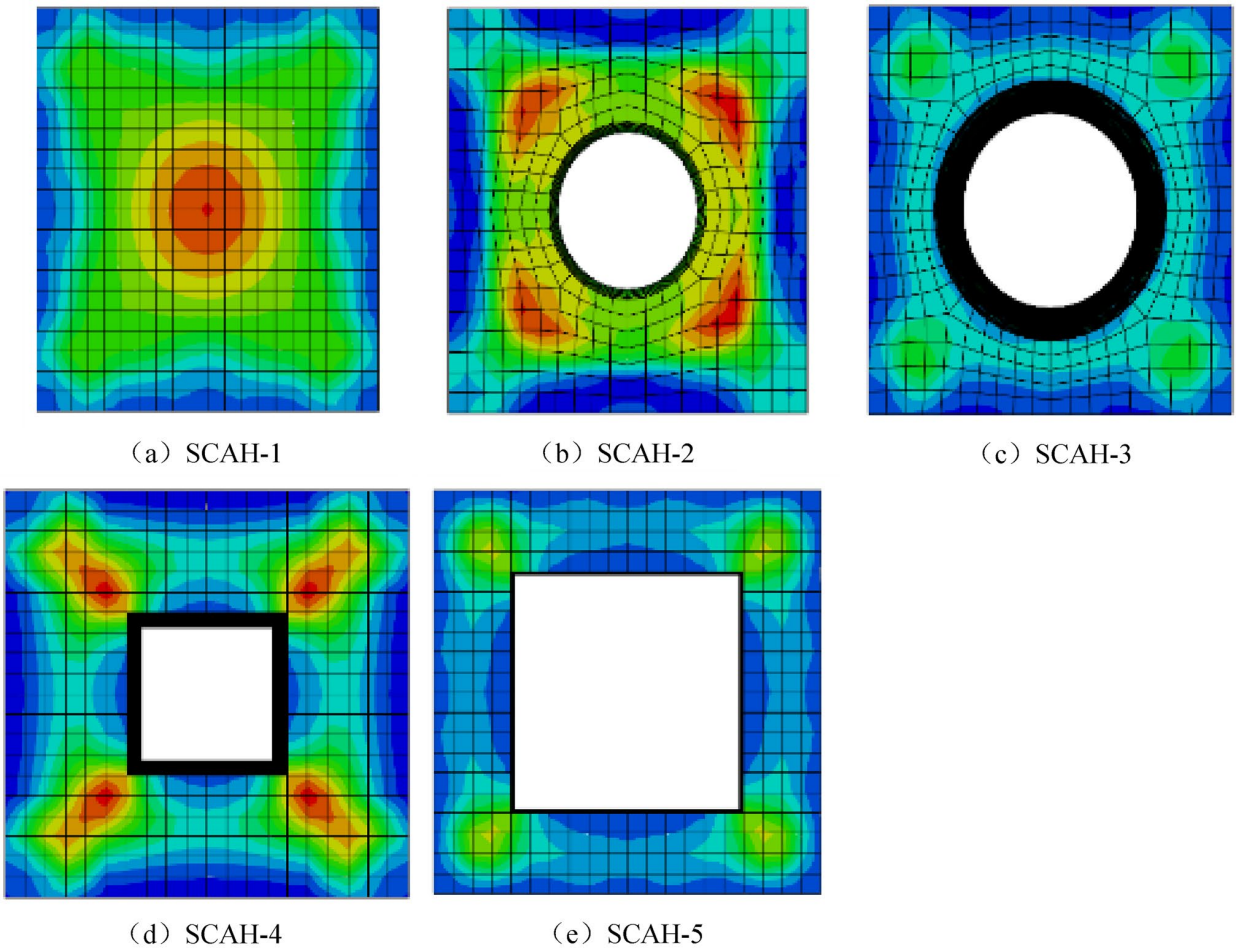
Simulation results of concrete restraint state of cross section of specimen (ABAQUS 2016 https://www.3ds.com/products-services/simulia/).

It can be seen from Fig. 24 that the concrete in the core area of specimens with small void ratio (SCAH-2, SCAH-4) can be regarded as triaxial constraint, and the concrete in the core area of specimens with large void ratio (SCAH-3, SCAH-5) can be regarded as biaxial constraint.

Mander model^28^ is established for stirrups restraining ordinary concrete square columns. The lateral restraint effect of stirrups on core concrete and “arch effect” of effective restraint area and rectangular restraint are considered. Considering whether the bearing capacity calculation of angle steel restrained high-strength lightweight aggregate concrete is applicable, the model is verified below:

For the specimen with small void ratio, the compressive strength formula of triaxial confined concrete is shown in Formula (4):

4 $$ f^{\prime}_{cc} = f^{\prime}_{c0} \left( {2.254\sqrt {1 + \frac{{7.94f^{\prime}_{l} }}{{f^{\prime}_{c0.} }}} - \frac{{2f^{\prime}_{l} }}{{f^{\prime}_{c0} }} - 1.254} \right). $$

For the specimen with large void ratio, the compressive strength formula of biaxial confined concrete is shown in Formula (5):

5 $$ f^{\prime}_{cc} = - 2.75\frac{{f^{{\prime}{2}} }}{{f^{\prime}_{c0} }} + 1.835f^{\prime}_{l} + f^{\prime}_{c0} . $$

Note: the test specimen with hollow ratio less than 16% is considered as small hollow ratio, otherwise it is the specimen with large hollow ratio.

In the formula: $f^{\prime}_{cc}$ represents compressive strength of confined concrete; $f^{\prime}_{c0}$ represents peak stress of unconstrained concrete, this paper takes $f^{\prime}_{c0} = 0.85f_{c}$^29^; $f^{\prime}_{l}$—lateral restraint stress.

6 $$ f_{l}^{\prime } = k_{e} \cdot f_{l} , $$

7 $$ k_{e} = 0.26\sqrt {\left( {\frac{{b_{c} }}{s}} \right)\left( {\frac{{b_{c} }}{{s^{\prime}}}} \right)\left( {\frac{1}{{f_{l} }}} \right)} k_{e} = 0.26. $$

**Figure 25:**
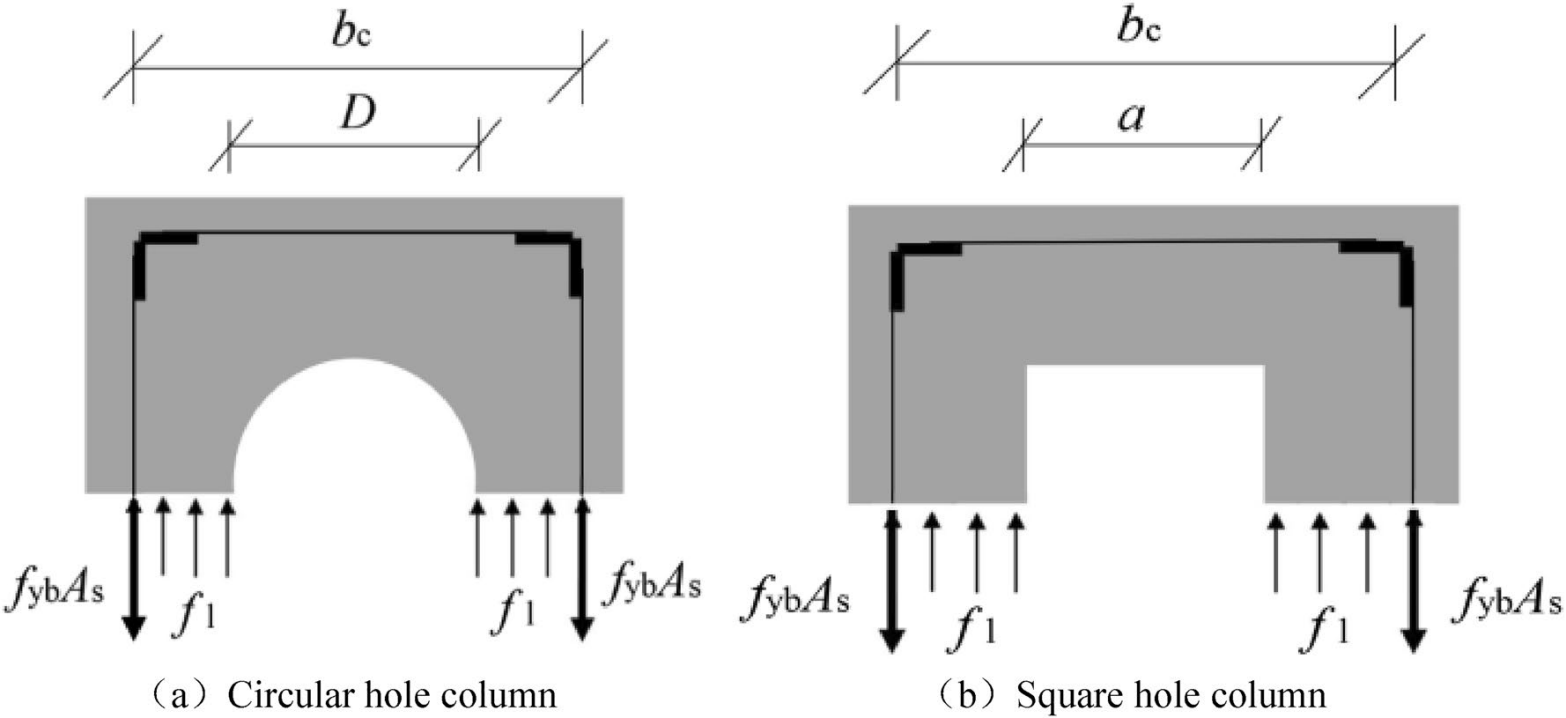
Schematic diagram of constraint stress calculation (Microsoft PowerPoint 2019 https://www.microsoft.com/zh-cn/microsoft-365/powerpoint).

In the formula: $k_{e}$ represents the effective constraint coefficient;$f_{l}$ represents the confining pressure on the batten plate, referring to the maximum restraint stress of the concrete in the core area when the batten plate yields;$b_{c}$ represents distance between batten centerlines; $s$ and $s^{\prime}$ represent the spacing of angle steel and batten respectively; According to the balance of force (as shown in Fig. 25), the confining pressure $f_{l}$ on the batten plate is calculated. The confining pressure $f_{l}$ on the batten plate of hollow column with round hole is as shown in formula (8), and the confining pressure $f_{l}$ on the batten plate of hollow column with square hole is as shown in formula (9).

8 $$ f_{l} = \frac{{2f_{yb} A_{sb} }}{{s^{\prime}(b_{c} - D)}}, $$

9 $$ f_{l} = \frac{{2f_{yb} A_{sb} }}{{s^{\prime}(b_{c} - a)}}. $$

In the formula: $f_{yb}$ and $A_{sb}$ represent the yield strength of the batten and the area of a single batten respectively;$D$ and $a$ represent diameter of round hole and side length of square hole. The cross-sectional area $A_{c}$ of core concrete of hollow column with round hole is shown in formula (10), and the cross-sectional area $A_{c}$ of core concrete of hollow column with square hole is shown in formula (11).

10 $$ A_{c} = b_{c}^{2} - \frac{{\pi D^{2} }}{4}, $$

11 $$ A_{c} = b_{c}^{2} - a^{2} . $$

Based on the superposition principle, the axial compression bearing capacity of composite hollow column of steel fiber, high-strength lightweight aggregate concrete and angle steel can be calculated according to formula (12):

12 $$ N_{u} = 0.9\delta \;(f^{\prime}_{cc} A_{c} + f^{\prime}_{c0} A_{cor} + f_{s} A_{s} ). $$

In the formula: $f^{\prime}_{cc}$ represents compressive strength of confined concrete; $f^{\prime}_{c0}$ and $A_{cor}$ represent the peak stress of unconstrained concrete and the cross-sectional area of concrete cover respectively; $f_{s}$ and $A_{s}$ represent the yield strength and total cross-sectional area of angle steel respectively.

Since the test results may be affected by the defects in the test loading, it is necessary to increase the reduction factor of 1.2. According to Table 6, it can be found that $P_{u}$/$N_{u}$ value is between 1.00 and 1.28 with the average value of 1.156, the standard deviation of 0.406 and the coefficient of variation of 0.351 when the superposition principle of Mander model is used for calculation and the influence of angle steel skeleton on concrete strength is considered. It is safer to calculate with the method for the calculated value is less than the test value.

**Table 6:** Comparison between test value and calculated value of bearing capacity of test column.

Specimen name	Test value $P_{u}$ (kN)	Confined concrete strength (MPa)	Strength of concrete cover (MPa)	Calculated $N_{u}$ (kN)	$P_{u}$/$N_{u}$
SCAH-1	2659.0	46.3	41.2	2324	1.14
SCAH-2	2480.7	48.4	41.2	2048.7	1.21
SCAH-3	1814.0	43.9	41.2	1583	1.15
SCAH-4	2590.2	48.1	41.2	2022.8	1.28
SCAH-5	1502.7	43.7	41.2	1500.6	1.00

## Conclusions

The influence of different void ratios and opening methods on the axial compressive properties of composite hollow column of steel fiber, high-strength lightweight aggregate concrete and angle steel is researched in the paper. Combining lightweight aggregate concrete with hollow column, the dead weight of the specimen is greatly reduced, and the finite element simulation is conducted. The bearing capacity of SCAH is calculated by three methods, and the following conclusions may be drawn:

1. The ends of SCAH-2–SCAH-5 are damaged severely, the cracks extends from the ends to the middle of the specimen, and the width increases. The column end of SCAH-1 is well constrained, and the failure of the specimen is mainly focused in the middle.
2. The peak load of composite hollow column of steel fiber, high-strength lightweight aggregate concrete and angle steel decreases significantly and the ductility increases along with the increase of void ratio; Under the same void ratio, the deformation ductility coefficient of hollow column with round hole is slightly lower than that of hollow column with square hole; The peak load of round hole is higher than that of square hole under large void ratio; Compared with the solid column, the hollow column with square hole at the hollow ratio of 16% has no great influence on the bearing capacity and ductility, and the ductility is better than the solid column.
3. The bearing capacity and load-longitudinal deformation curves obtained by finite element simulation are in good agreement with the experimental values. In the elastic stage, the compressive stress of concrete is less than − 20 MPa, and the angle steel and batten plate do not reach the yield stress; In the elastic–plastic stage, the maximum compressive stress of the concrete core area of the specimen with small void ratio increases significantly (up to − 60 MPa), while the compressive stress of the concrete of the specimen with large void ratio increases slightly (about − 30 MPa), and the yield stress is reached in the full length of the angle steel, but the batten still does not reach the yield stress; In the descending section (BC), the compressive stress of the concrete surface of the specimen is small (about − 6 to − 13 MPa), and even a small tensile stress (0–8 MPa) occurs. The radial deformation of the concrete increases rapidly, and some battens reach the yield stress.
4. The influence of angle steel skeleton on concrete strength is considered in Mander model, and then the superposition calculation is conducted. It is safer to calculate with the method for the calculated value is less than the test value, and the calculation accuracy is high.

## Data Availability

The datasets generated and/or analysed during the current study are not publicly available due fund confidentiality but are available from the corresponding author on reasonable request.
